# Recurrent Hypokalemia and Adrenal Steroids in Patients With APECED

**DOI:** 10.3389/fendo.2022.904507

**Published:** 2022-06-22

**Authors:** Joonatan Borchers, Outi Mäkitie, Jarmo Jääskeläinen, Saila Laakso

**Affiliations:** ^1^ Children’s Hospital, Pediatric Research Center, University of Helsinki and Helsinki University Hospital, Helsinki, Finland; ^2^ Folkhälsan Research Center, Folkhälsan Institute of Genetics, Helsinki, Finland; ^3^ Research Program for Clinical and Molecular Metabolism, Faculty of Medicine, University of Helsinki, Helsinki, Finland; ^4^ Department of Molecular Medicine and Surgery, Karolinska Institutet, and Clinical Genetics, Karolinska University Hospital, Stockholm, Sweden; ^5^ Kuopio Pediatric Research Unit, University of Eastern Finland and Kuopio University Hospital, Kuopio, Finland

**Keywords:** APS-1, primary adrenal insufficiency (PAI), potassium, adrenal androgens, DHEA

## Abstract

**Context:**

Hypokalemia is a common finding in patients with autoimmune polyendocrinopathy-candidiasis-ectodermal dystrophy (APECED) but its exact cause often remains unknown.

**Objective:**

To explore the prevalence and etiology of hypokalemia and the role of adrenal steroids therein in a cohort of patients with APECED.

**Methods:**

We performed a cross-sectional assessment and retrospective data collection on 44 Finnish patients with APECED to identify subjects with episodes of hypokalemia. Also 68 healthy matched controls attended the cross-sectional evaluation. Factors associating with a tendency for hypokalemia were analyzed by reviewing medical records during 1960-2021 and performing a cross-sectional analysis of serum adrenal steroids.

**Results:**

In total 14 of the 44 APECED patients (32%) had episodes of hypokalemia; 2 presented with hypokalemia at cross-sectional evaluation and 12 had a history of hypokalemia before the cross-sectional evaluation. Hypokalemic episodes started at the median age of 14.1 years; 12/14 (86%) had primary adrenal insufficiency (PAI). The median number of hypokalemic periods per year was 0.3 (range 0.04-2.2); the frequency correlated positively with the number of clinical APECED manifestations at the time of cross-sectional evaluation (r=0.811, p<0.001). Etiologies of hypokalemia varied but episodes often occurred when new clinical manifestations developed and during hospitalizations. Three patients had kidney defects, also associated with electrolyte imbalances. Severity of hypokalemia varied (range 2.2-3.2 mmol/L), but no severe complications were observed. At cross-sectional evaluation, patients with PAI (n = 30) had significantly lower median plasma potassium and higher sodium concentration than controls, suggesting that fludrocortisone treatment contributed to hypokalemia. Detailed analysis of adrenal steroids provided no conclusive differences between patients with and without episodes of hypokalemia.

**Conclusions:**

In APECED, hypokalemia is common and varies in terms of frequency, etiology, and severity. PAI and kidney disease predispose to hypokalemia. In addition, hypokalemic periods seem to be more common in patients with more severe phenotype of APECED.

## 1 Introduction

Primary adrenal insufficiency (PAI) is a major clinical manifestation of autoimmune polyendocrinopathy-candidiasis-ectodermal dystrophy (APECED), also known as autoimmune polyendocrine syndrome type I (APS-I). Among patients with APECED, PAI is often diagnosed during childhood. However, it may present at various ages, the age of onset ranging from 2 to 55 years (1–5). Prevalence of PAI varies from 55% to 84% in different cohorts, although this variation may partly be explained by variable length of follow-up (1–6). Depending on the population, PAI is the first manifestation of APECED in 0-21% of patients (1, 2, 5, 6).

Abnormal concentrations of mineralocorticoids or glucocorticoids may cause electrolyte disturbances in patients with APECED. In some patients, hypokalemia is accompanied with high blood pressure with or without increased concentrations of mineralocorticoids. This has been seen in both patients with and without PAI (3, 7). In addition, some patients with PAI have exceptionally high sensitivity to mineralocorticoid replacement therapy. These patients require potassium substitution and sometimes potassium sparing diuretics to normalize the electrolyte levels and blood pressure (7). Apart from a single case with co-existing renal tubulopathy (8), no studies have reported hypokalemia in cohorts of patients with PAI due to other causes than APECED indicating that other factors than PAI alone may contribute to hypokalemia in patients with APECED.

In this study, we aimed to describe characteristics of hypokalemic periods and their relation to the clinical course of PAI and adrenal steroid profile in the Finnish APECED cohort, which has been carefully followed for over 50 years. We performed a cross-sectional assessment and retrospective data collection of 44 Finnish patients with APECED to identify subjects with episodes of hypokalemia. The cross-sectional evaluation included also 68 healthy matched control subjects. Factors associating with tendency for hypokalemia were subsequently analyzed by reviewing medical records and by performing a cross-sectional analysis of serum adrenal steroids.

## 2 Materials and Methods

### 2.1 Subjects

For the study visits during 2015-2016, we invited all Finnish patients with APECED (1, 9). Age-, gender-, and ethnicity-matched healthy adult control subjects were identified through population register and included as described previously (9). Control subjects with daily medication affecting bone, immunity, or hormones were excluded. For the seven pediatric patients aged 7–16.5 years, no control subjects were recruited. An ethical approval was obtained from the Research Ethics Committee of the Hospital District of Helsinki and Uusimaa. Informed written consent was obtained from all study participants and for subjects aged <18 years, also from their guardians.

### 2.2 Clinical Assessment and Patient Register Data

In the cross-sectional part of the study, during the study visit we measured, height, weight, and blood pressure of all patients and control subjects. Participants filled a questionnaire that included information on lifestyle factors, such as diet. From medical and study records, we retrospectively collected the date of diagnosis of clinical manifestations and the date of initiation of medications. The following manifestations were included: chronic mucocutaneous candidiasis, hypoparathyroidism (HP), PAI, diabetes, hypogonadism, hypothyroidism, growth hormone deficiency, hepatitis, intestinal dysfunction, exocrine pancreatic insufficiency, tubulointerstitial nephritis (TIN), alopecia, vitiligo, keratopathy, enamel dysplasia, and rash with fever.

**Figure 1:**
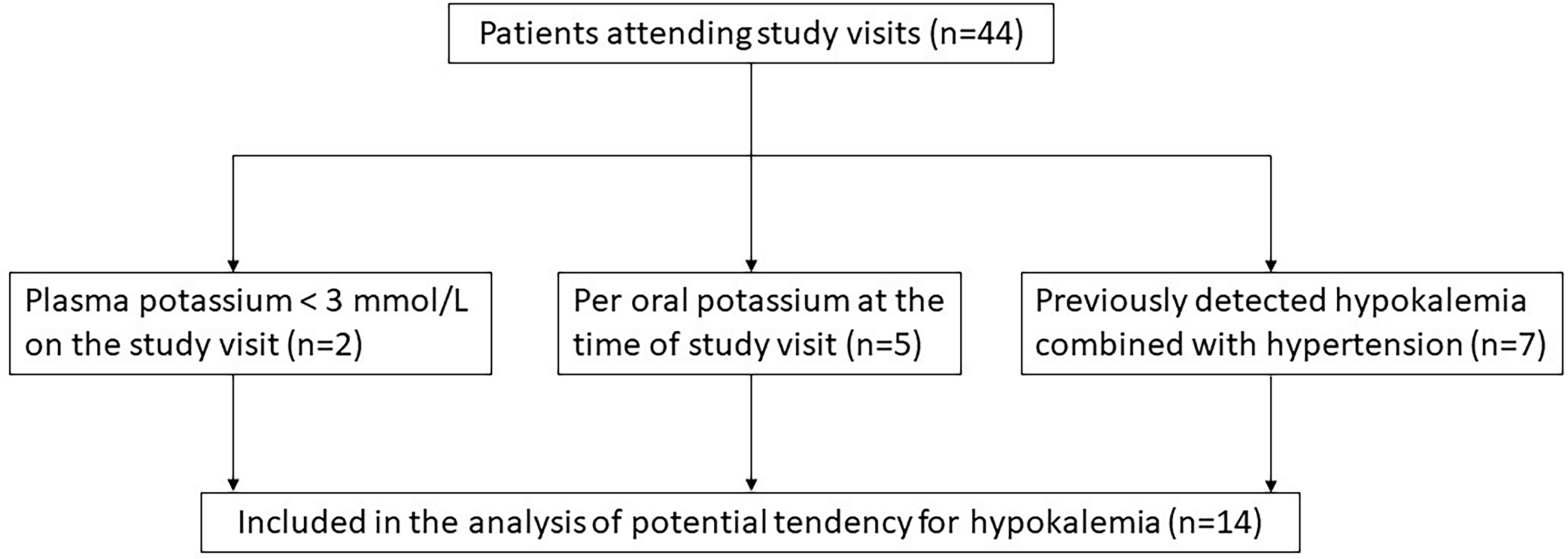
Selection of patients with potential tendency for hypokalemia among 44 patients with APECED.

Patients with a tendency for hypokalemia were identified using the following criteria: 1) hypokalemia (P-K < 3.0 mmol/l) measured at study visit, 2) use of potassium supplement at the time of study visit, or 3) previously diagnosed hypokalemia-hypertension (**Figure 1**). From the patient records of the identified patients (n=14), all available measurements of sodium and potassium concentrations, dosages of glucocorticoid, fludrocortisone, potassium supplement, and spironolactone treatment, as well as the time and cause of hospitalizations were collected. The follow-up time started from the date when the first electrolyte concentrations were available and ended at the date of last available electrolyte concentrations. The follow-up periods ranged from June 1960 to September 2021. As laboratory methods had changed during decades, the lower boundary of reference range was defined as the lowest concentrations of all reported reference ranges (3.3 mmol/L) and upper boundary of reference range as the highest concentrations of all reported reference ranges (5.0 mmol/L). A hypokalemic period was defined as the period from the first hypokalemic to next normokalemic measurement.

### 2.3 Biochemical Measurements in the Cross-Sectional Study

Blood samples and second void urine were collected between 07:00 and 10:00 h after an 8–12-h fast in both patients and control subjects before the morning medication. Plasma and urine sodium, potassium, and creatinine as well as urine alpha-1-microglobuline (U-A1Miglo) were measured using standard methods at the University Hospital’s core laboratory HUSLAB. Dehydroepiandrosterone sulfate (DHEAS) was measured with standard methods at HUSLAB using chemiluminescent immunoassays [Architect (Abbott Laboratories) and Atellica IM (Siemens)] and appropriate coefficient was used to achieve comparability of the results.

Serum cortisol, cortisone, and dehydroepiandrosterone (DHEA) were extracted with liquid-liquid extraction using diethyl ether. Samples were analyzed on a liquid chromatography-tandem mass spectrometry system LC-MS/MS system equipped with a TQ-5500 mass spectrometer (Sciex, MA, US) and an Agilent series 1200 HPLC system with a binary pump (Waldbronn, Germany). The LC-MS/MS method for cortisol had a limit of quantitation (LoQ) of 0.5 nmol/L; inter-assay coefficient of variations (CV%) was 5.7%. LoQ for cortisone was 0.3 nmol/L; inter-assay CV% was 8.3. LoQ for DHEA was 0.5 nmol/L; inter-assay CV% was 5.5%. Serum aldosterone concentrations were measured by LC-MS/MS using dichlormethane extraction. LC-MS/MS system was equipped with an TQ-5500 mass spectrometer (Sciex, MA, US) and an Agilent series 1200 HPLC system with a binary pump (Waldbronn, Germany). The LC-MS/MS method for aldosterone had a LoQ of 20 pmol/L; inter-assay CV% was 5.5%. Androstenedione concentrations were measured as described previously (10).

21-hydroxylase (21OH) autoantibodies and side-chain cleavage enzyme (SCC) autoantibodies were measured with radio-ligand binding assay as described previously (11).

### 2.4 Statistical Analysis

The results are presented as median (range). Mann-Whitney U test was used to test differences between groups and Spearman’s Rho for correlations. Highest value below LoQ was used for the results that were less than the quantitation limit. Statistical analysis was carried out using IBM SPSS Statistics (version 25.0) and GraphPad Prism (version 9).

## 3 Results

### 3.1 Patient Characteristics

The cohort comprised 44 patients (27 females) with a median age of 37.8 years (7.0-70.1 years). Of the 44 patients, seven (five females) were under 18 years of age [median (range), 12.5 (7.0-16.5)] and five of them had PAI.

All patients carried known pathogenic variants of the AIRE gene (NM_000383.4). All of them harbored at least one copy of the Finnish founder mutation c.769C>T, p.Arg257Ter in the AIRE. Of the 44 patients, 77% were homozygous for this pathogenic variant while in the others it was compounded with c.967_979del13, (p.Leu323fs) (n = 5), c.932G>A, (p.Cys311Tyr) (n = 2), or c.891C>A, (p.Asp297Glu), (n = 2). PAI had been diagnosed in 36 patients (81%). PAI was the first manifestation in two patients (5%) and the first endocrinopathy in 14 patients (32%). Of the 36 patients with PAI, glucocorticoid replacement therapy was initiated at median age of 10.4 years (5.4-34.8) and mineralocorticoid replacement was initiated at median age of 10.4 years (5.4-40.2). DHEA replacement therapy was used at least periodically in eight (18%, one male) patients, and it was initiated at the median age of 33.6 years (14.4-52.9).

### 3.2 Patients With Hypokalemia

Occasional low concentrations of potassium were seen in many patients with APECED. However, some patients had recurrent periods of hypokalemia and fluctuating electrolyte concentrations. Of the 44 patients attending the study visits, two patients had current potassium concentration lower than 3.0 mmol/L during the study visit (**Figure 1**). Of the remaining 42 patients, five patients were using daily potassium substitution for hypokalemia but without combined hypertension at the time of the study visit. All five patients had PAI and one of them had also TIN with kidney transplantation performed over 25 years before study visit and cyclosporine as immunosuppressive treatment at the time of study visit. This patient did not need potassium substitution before the kidney transplantation, but it was initiated shortly after the transplantation. In addition, hypokalemia combined with hypertension had previously been detected in seven patients. Of these seven patients, five had PAI and of the two without PAI one had TIN and the other had renal tubular acidosis (RTA). The patient with TIN had undergone kidney transplantation a year before the study visit and was using tacrolimus and mycophenolate mofetil. This patient had been using per oral potassium substitution prior the kidney transplant. The patient with RTA was treated with sodium bicarbonate as well as per oral potassium substitution. Thus, we retrospectively studied the electrolyte concentrations of 14 (10 females, 71%) patients with APECED (**Figure 1**). Altogether 86% of them (12/14) had PAI and had used fludrocortisone replacement therapy at least periodically.

The selected 14 patients had median of 9.5 (1-61) hypokalemic periods during median of 34.3 years (14.3-53.1) of follow-up. Age at the time of hypokalemic period ranged from 6.7 to 62.6 years. Median number of hypokalemic periods per year was 0.3 (0.04-2.2). In addition, the number of hypokalemic periods per year correlated positively with the number of clinical manifestations of APECED at the time of cross-sectional evaluation (r=0.811, p<0.001), suggesting that more severe clinical picture of APECED may increase the risk for hypokalemia. Details on the hypokalemic periods are shown in **Table 1**.

**Table 1:** Table showing details on hypokalemic periods (HypoK) in 14 patients with APECED.

Patient	Age at 1. HypoK (y)	Follow-up (y)	PAI	TIN	Diarrhea	P <3	P 3-3.2	HypoK/y	P substit.	Spironol.	No. of manifestations	No. of HypoK during hospital care	
												All HypoK during hospital care	HypoK upon admission
1	6.7	31.4	Y	N	E	4	7	0.4	–	–	11	3	3 (100%)
2	6.8	27.7	Y	N	–	32	29	2.2	42 (68%)	25 (40%)	12	9	6 (67%)
3	6.8	21.6	N	Y*	E/exo	3	6	0.4	7 (78%)	–	12	4	3 (75%)
4	7.2	37.1	Y	N	E/exo	5	37	1.1	26 (62%)	–	13	8	2 (25%)
5	7.4	14.3	Y	N	E	–	4	0.3	4 (100%)	–	6	3	3 (100%)
6	13.2	23.2	Y	N	–	3	5	0.3	–	–	8	0	–
7	13.8	39.4	Y	N	E	5	6	0.3	1 (9%)	–	10	4	3 (75%)
8	14.5	22.8	N	Y	E	5	5	0.4	8 (80%)	3 (30%)	9	0	–
9	15.4	41.4	Y	N	E	2	12	0.3	2 (14%)	–	9	3	3 (100%)
10	19.9	24.9	Y	N	C	–	1	0.04	–	–	7	1	1 (100%)
11	23.6	53.1	Y	Y	–	7	5	0.2	9 (75%)	3 (30%)	8	2	1 (50%)
12	29.0	50.1	Y	N	–	–	4	0.1	1 (25%)	–	8	0	–
13	34.9	41.4	Y	N	C/exo	–	2	0.1	1 (50%)	–	8	0	–
14	42.2	44.8	Y	N	–	–	4	0.1	–	–	6	0	–
All median (range)	14.1 (6.7-42.2)	34.3 (14.3-53.1)	12	3		3 (0-32)	5 (1-37)	0.3 (0.04-2.2)	2 (0-42)		8.5 (6-13)	3 (0-9)	2 (0-6)

Patients with primary adrenal insufficiency (PAI) and tubulointerstitial nephritis (TIN) are shown. In addition, patients with chronic (C) or episodic (E) diarrhea and/or exocrine pancreatic insufficiency (exo) are presented. Periods of mild hypokalemia (P 3-3.2) and more severe hypokalemia (P<3) are shown separately and total number of hypokalemic periods per year (HypoK/y) is presented. Also, the proportion of hypokalemic periods when using per oral potassium substitution (P-substit.) and spironolactone as well as number of clinical manifestations in the end of follow-up (No. of manifestations) are presented. Median (range) of all patients are presented at the bottom.

Y, yes; N, no. *Patient had renal tubular acidosis.

**Figure 2:**
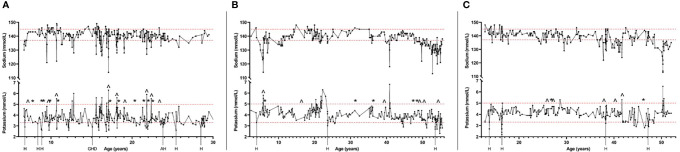
Timelines of three patients with APECED and tendency for hypokalemia showing the fluctuating serum concentrations of sodium and potassium during follow-up of the patients. Also, hospitalizations (H) and other stressful periods of disease course at the time of hypokalemia are shown. Changes of fludrocortisone replacement dosage of 50% or more are also shown (^, increase of the dosage; *, decrease of the dosage). Figure shows one patient selected by different criteria for tendency of hypokalemia: Patient **(A)** had been previously detected with hypokalemia combined with hypertension; Patient **(B)** was using daily potassium substitution at the time of study visit; Patient **(C)** had current potassium level lower than 3.0 mmol/L during the study visit. Dashed lines show the reference range of potassium (3.3-5.0 mmol/L) and sodium (137-145 mmol/L). H, Hospital care; GHD, Growth hormone deficiency detected; AH, Activation of autoimmune hepatitis.

Electrolyte balance seemed to fluctuate in periods. In most of the patients, there were long periods when the electrolyte levels were stable, and no significant electrolyte disturbances were detected. In between of these stable periods, there were periods for several months when the electrolyte concentrations fluctuated considerably in short intervals and electrolyte imbalances were seen repeatedly (**Figure 2**). These periods of imbalanced electrolyte concentrations were often seen during challenging and stressful periods of disease course, such as development of new APECED manifestations, depression or other mental illness, or during hospitalizations.

Of the 14 patients with hypokalemic episodes, nine (64%) patients had at least one episode of hypokalemia during hospitalizations (**Table 1**). Of these nine patients, eight had PAI. In most of the hypokalemic episodes during hospital care, the hypokalemia was seen already when the patient was admitted to the hospital (71%, **Table 1**). Infection was the etiology for at least one hospitalization in three patients. Of these three patients, two had at least one episode of gastroenteritis and diarrhea as the cause for hospitalization. Other causes for hospitalization included hypocalcemia, development of new clinical manifestations of APECED, abdominal pain, and other unspecified general weakness. In comparison, hyperkalemia was reported in seven of these same patients (7/14, 50%), during at least one hospitalization separately from the hypokalemic episode. All seven patients had PAI. Hyperkalemia was present already when the patient was admitted to the hospital in 81% of cases. During hospital stay, patients with PAI often had increased dosage of per oral or intravenous glucocorticoid replacement therapy, dosage of fludrocortisone replacement was not increased. No electrolyte imbalance-induced arrythmias requiring treatment were reported.

Even though increasing the fludrocortisone dosage often decreased potassium level, it rarely led to hypokalemia. However, the impact of the changed dosage of fludrocortisone varied even in an individual patient (**Figure 2**). In some situations, even a small adjustment led to major changes in the electrolyte concentrations but in other situations increasing or decreasing the dosage by 100% had no notable impact. This was seen in both children and adults. Fludrocortisone substitution was also modified by the patients themselves because of symptoms such as weight gain combined with edema, fatigue, or salt craving.

In addition to decreasing the dosage of fludrocortisone, hypokalemia was often managed with per oral potassium substitution. Per oral potassium was used in 12 (12/14, 86%) patients at least occasionally during the follow-up. It was the first treatment for hypokalemia in both patients without PAI. The potassium dosage varied between 750-10000 mg/day. Four patients (29%, one without PAI) used spironolactone combined with potassium substitution during the follow-up period with doses varying between 12.5-100 mg/day. In three patients, spironolactone was initiated when hypokalemia was combined with hypertension. It was initiated at the age of 14.5-54.0 years. Ketoconazole [which may block adrenal steroidogenesis by inhibiting several CYP450 enzymes (12, 13)] was used regularly in one patient and as prophylactic treatment in one patient. However, no notable fluctuations in potassium concentrations during the short treatment periods with ketoconazole were found in these patients. High consumption of salted liquorice has also been reported in association with hypokalemia (7, 14). No high consumption of salted liquorice was reported in this cohort of patients. Of the 14 patients, two patients reported occasional increased craving for salt. No special diets concerning salt intake was reported by the patients.

Of the 14 patients with hypokalemic episodes, nine (64%) had been reported to suffer from chronic (n = 2) or episodic diarrhea (n = 7; **Table 1**). In addition, three of them with diarrhea had been diagnosed with exocrine pancreatic insufficiency. For comparison, in other patients that did not have tendency for hypokalemia, diarrhea was reported in 15 patients (50%) from which three had chronic diarrhea and 12 had episodic diarrhea. Exocrine pancreatic insufficiency was diagnosed on two of them.

### 3.3 Relationship Between Potassium and Adrenal Steroids

We then assessed the association between electrolyte balance and adrenal steroid profiles based on the data collected at the cross-sectional study visits in the patients with APECED and controls. Of the 44 patients attending the study visit, two patients were excluded from adrenal steroid analysis based on use of medications (one PAI patient had taken morning medications before blood sampling and one non-PAI patient was on daily prednisolone medication due to keratitis). For the comparison with adult control subjects, we excluded patients under 18 years of age. Of the remaining 35 adult patients, 30 had PAI; 25 (83%) of them received hydrocortisone replacement therapy and five (17%) received other cortisone medication alone (prednisone, n=2; prednisolone, n=1) or prednisolone combined with hydrocortisone (n=2). The characteristics of adult patients with and without PAI, and control subjects are presented in **Table 2**.

**Table 2:** Table showing characteristics and measurements taken at the study visits of adult patients with primary adrenal insufficiency (PAI) due to APECED, patients with APECED without PAI (non-PAI), and control subjects.

Characteristic	PAI	non-PAI	Controls
No. of patients (female %)	30 (60%)	5 (60%)	68 (63%)
Age (years)	44.0 (19.3-70.1)	39.1 (23.1-62.7)	48.6 (21.5-72.7)
Number of manifestations	7 (4-12)	7 (4-12)	–
Weight (kg)	62.4 (37.7-95.5)	62.9 (49.6-65.7)	73.5 (49.3-159.2)
Height (cm)	167 (152-189)	171 (162-175)	170 (150-190)
Systolic BP (mmHg)	132 (106-174)	131 (113-151)	128 (109-189)
Diastolic BP (mmHg)	84 (67-98)	77 (70-100)	83 (53-119)
Hypoparathyroidism, n (%)	25 (83%)	4 (80%)	–
21OH autoantibodies, n (%)	17 (57%)	3 (60%)	–
Hydrocortisone equivalent* (mg/d) (n=30)	20 (10-35)	–	–
Fludrocortisone (mg/d) (n=29)	0.1 (0.025-0.5)	–	–
Potassium (mg/d) (n=10)	1250 (426-6000)	–	–
DHEA (mg/d) (n=6)	16 (6-50)	–	–

Results are presented as median (range) unless otherwise specified.

BP, blood pressure; 21OH, 21-hydroxylase; DHEA, dehydroepiandrosterone. *Hydrocortisone in 83%; other cortisone medication in 17%.

#### 3.3.1 Adult Patients vs. Controls

**Figure 3:**
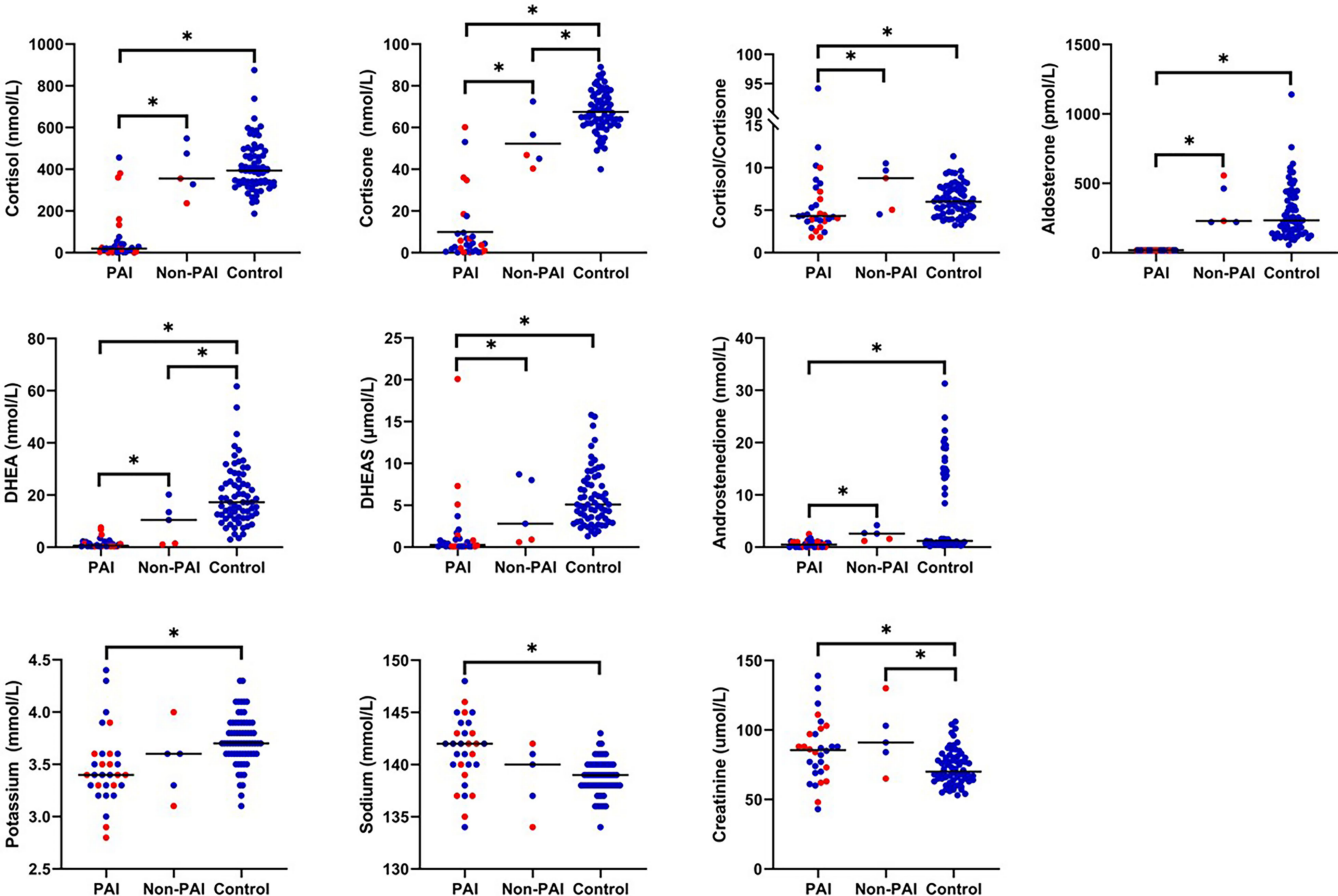
Concentrations of adrenal steroids and electrolytes in adult patients with PAI due to APECED (n=30), adult patients with APECED without PAI (Non-PAI, n=5), and healthy adult controls (n=68). Fourteen patients with tendency for hypokalemia are marked in red. *p < 0.05.

Potassium concentrations were significantly lower and sodium concentrations higher in patients with PAI compared with control subjects (**Figure 3**). Patients with PAI had significantly lower concentrations of both cortisol and cortisone as well as cortisol/cortisone ratio compared with control subjects. Also, aldosterone, DHEA, DHEAS, and androstenedione were all significantly lower in patients with PAI compared with the two other groups. Creatinine concentrations were significantly higher in patients with PAI [85.5 µmol/L (43-139); p=0.003] compared with control subjects [70 mmol/L (53-106)] (**Figure 3**). In patients with PAI, potassium concentrations did not correlate with plasma renin concentrations (r = 0.293, p=0.116). Neither did dosage of fludrocortisone correlate with renin (r = 0.035, p=0.853) or potassium (r = -0.302, p=0.105) concentrations.

In patients without PAI, electrolyte concentrations did not differ from control subjects but significant differences were observed between adrenal steroid profiles. Patients without PAI had significantly lower concentrations of cortisone compared to control subjects [46.9 nmol/L (40.4-72.5); 66.5 nmol/L (40.0-89.0), p=0.011]. In addition, concentration of DHEA was significantly lower in patients without PAI [10.5 nmol/L (1.1-20.2)] compared with controls [17.3 (3.1-61.7), p=0.036]. Concentrations of cortisol, aldosterone, DHEAS, androstenedione, or cortisol/cortisone ratio did not differ between patients without PAI and controls. Patients without PAI had also significantly higher creatine concentrations [91 mmol/L (65-130); p=0.023] than control subjects (**Figure 3**).

Of the seven patients without PAI in the entire cohort, three had DHEA concentrations below the reference range; all three patients had autoantibodies against 21OH and none against SCC. Of these three patients, one patient under 16 years of age developed mineralocorticoid deficiency at 0.1 years after the study visit, and one adult patient developed glucocorticoid deficiency at 0.1 years after the study visit.

#### 3.3.2 Hypokalemia Patients vs. Other Patients

Of all 42 patients, one patient under 18 years of age with PAI but no hypokalemic tendency was excluded from the analysis because of end stage kidney disease due to TIN. Therefore, 41 patients were included in this analysis.

Current electrolyte and adrenal steroid concentrations did not differ in patients with a tendency for hypokalemia compared to all other patients or patients with PAI without hypokalemic tendency (**Table 3**). However, when patients with a tendency for hypokalemia were compared to patients without PAI, significantly lower concentrations of cortisol, cortisone, cortisol/cortisone-ratio, and DHEA were found in patients with hypokalemic tendency, which reflected the higher proportion of patients with PAI in the hypokalemic group. Patients without PAI had significantly higher ratio of urine potassium/creatinine compared to patients with hypokalemic tendency. No other differences were found in urine potassium/creatinine or sodium/creatinine ratios between the groups. No difference was found in concentrations of creatinine or U-A1Miglo. Concentrations of potassium and renin did not correlate in patients with a tendency for hypokalemia (r = -0.181, p=0.536). Neither did dosage of fludrocortisone correlate with potassium (r = -0.338, p=0.237) or renin (r = 0.301, p=0.296) concentrations in these patients. Finally, no differences were found in adrenal steroid or electrolyte concentrations between patients with earlier detected hypokalemia combined with hypertension and other patients (data not shown).

**Table 3:** Table showing characteristics, and concentrations of adrenal steroids and electrolytes measured at the study visits of 41 patients with APECED: separately for patients with hypokalemic tendency (HypoK, 12 patients with PAI), other patients with PAI, other patients without PAI (non-PAI) and other patients with and without PAI together (PAI + non-PAI).

Characteristic	HypoK	PAI	non-PAI	PAI + non-PAI
No. of patients (female %)	14 (71%)	22 (50%)	5 (80%)	27 (56%)
No. of manifestations	8 (6-12)	6 (3-11)**	5 (1-7)**	6 (1-11)**
Age (years)	40.2 (19.3-62.1)	38.9 (12.5-70.1)	39.1 (7.0-62.7)	39.1(7.0-70.1)
Weight (kg)	56.7 (37.7-95.5)	63.9 (40.0-93.4)	49.8 (17.6-64.7)	62.9 (17.6-93.4)
Height (cm)	167 (152-175)	166 (143-189)	162 (116-171)	166 (116-189)
BMI (kg/m²)	21.0 (15.0-40.7)	23.5 (17.7-36.6)*	21.2 (16.4-23.5)	22.8 (16.4-36.6)
Systolic BP (mmHg)	131 (106-154)	133 (112-174)	131 (96-151)	132 (96-174)
Diastolic BP (mmHg)	85.5 (67-96)	83 (68-98)	72 (59-100)	82 (59-100)
Chronic diarrhea n (%)	2 (14%)	3 (14%)	–	3 (11%)
Exocrine pancreas insufficiency n (%)	3 (21%)	1 (5%)	1 (20%)	2 (7%)
S-Cortisol (nmol/L) ^a^	26.9 (<0.5-380.0)	18.4 (<0.5-773.0)	464 (298.0-548.0)**	22.6 (<0.5-773.0)
S-Cortisone (nmol/L) ^b^	6.3 (<0.3-60.2)	3.6 (<0.3-55.7)	49.6 (45.1-72.5)**	4.4 (<0.3-72.5)
Cortisol/Cortisone	4.3 (1.8-10.0)	4.4 (2.1-94.2)	9.7 (4.5-10.5)*	4.5 (2.1-94.2)
S-Aldosterone (pmol/L) ^c^	19.0 (<20.0-557.0)	19.0 (All <20.0)	222.0 (<20.0-463.0)	19.0 (<20.0-463.0)
S-DHEA (nmol/L) ^d^	0.8 (<0.5-7.6)	0.4 (<0.5-3.5)	10.5 (<0.5-20.2)*	0.8 (<0.5-20.2)
S-DHEAS (µmol/L) ^e^	0.4 (<0.2-20.1)	0.1 (<0.2-3.7)	2.8 (<0.2-8.7)	0.3 (<0.2-8.7)
S-Androstenedione (nmol/L) ^f^	0.7 (<0.1-2.5)	0.5 (<0.1-1.7)	2.6 (0.2-4.2)	0.5 (<0.1-4.2)
P-Renin (mU/L) ^g^	38.5 (<5.0-420.0)	41.5(<5.0-1300.0)	29.0 (14.0-36.0)	32 (<5.0-1300.0)
P-Potassium (mmol/L)	3.4 (2.8-4.0)	3.4 (3.0-4.4)	3.6 (3.3-3.8)	3.4 (3.0-4.4)
P-Sodium (mmol/L)	142 (134-146)	142 (134-148)	140 (137-141)	141 (134-148)
P-Creatinine (µmol/L)	87 (48-130)	80 (43-139)	84 (32-103)	82 (32-139)
U-Potassium/U-Creatinine	4.5 (1.5-9.1)	3.5 (1.8-12.4)	6.0 (5.5-20.8)*	4.6 (1.8-20.8)
U-Sodium/U-Creatinine	11.6 (2.8-32.3)	12.6 (4.0-30.4)	9.1 (6.6-25.1)	11.5 (4.0-30.4)
U-A1Miglo increased (n)	1	3	–	3

Results of other groups are compared with HypoK-patients and significant differences are shown.

Results are presented as median (range) unless otherwise specified. BMI, body mass index; BP, blood pressure; S, serum; DHEA, dehydroepiandrosterone; DHEAS, dehydroepiandrosterone sulfate; P, plasma; U, urine; A1Miglo, alfa-1-microglobuline. **p < 0.01, *p < 0.05, ^a^below the level of quantification in 2, ^b^below the level of quantification in 4, ^c^below the level of quantification in 36, ^d^below the level of quantification in 21, ^e^below the level of quantification in 20, ^f^below the level of quantification in 16, ^g^below the level of quantification in 2.

## 4 Discussion

In this study we evaluated possible etiologies and characteristics of hypokalemia in patients with APECED. To obtain a more extensive understanding of the potential association of adrenal function and electrolyte balance in patients with APECED, we also compared adrenal steroid and electrolyte concentrations in patients with APECED and healthy controls. Our findings indicate that hypokalemic periods in patients with APECED vary highly in terms of frequency, etiology, and severity.

In this study, we found that recurrent hypokalemia is a relatively common finding with varying severity in patients with APECED. In addition to severity, also the specific cause for hypokalemia seems to vary and no single common etiology was found in our cohort of patients. Etiologies that were present in patients included changes in medications, severe illnesses as well as challenging periods of disease, and mental illnesses. Ketoconazole has been found to inhibit adrenal steroidogenesis (12, 13). However, in our retrospective setting, we did not find any clear association with electrolyte disturbances and ketoconazole treatment.

Age at the first hypokalemic period varied widely. However, most patients experienced the first hypokalemic periods during childhood. In our cohort we found a positive correlation between hypokalemic periods per year and number of clinical manifestations. This could indicate that hypokalemia is more common in patients with more severe clinical phenotype of APECED.

We found that hypokalemia during hospital care is a relatively common finding in patients with APECED. This is notable, since in patients with PAI, severe illnesses that require hospital care often lead to adrenal crisis and hyperkalemia (15). In fact, in our study we found that hypokalemia was more prevalent during hospital care than hyperkalemia. During hospital care, patients with PAI often had increased dosage of glucocorticoid replacement, which may affect the potassium concentration. Other medications that may affect the electrolyte balance, such as diuretics were also used occasionally during hospital care. However, majority of hypokalemic and hyperkalemic episodes were seen already when the patient was admitted to the hospital, indicating that the altered medication during hospitalization is not often the initial etiology for potassium imbalance.

Adjusting the dosage of fludrocortisone lower was a common measure to increase the concentration of potassium in all patients with PAI. However, we found that the impact of the change in fludrocortisone dosage varied in many patients. In our cross-sectional analysis, patients with PAI had significantly lower concentrations of potassium and higher concentrations of sodium compared to healthy control subjects. These findings could indicate overtreatment with fludrocortisone. However, the median dose of fludrocortisone in our patient cohort was 0.1 mg daily, which is a commonly recommended daily dose for patients with PAI (16, 17). On the other hand, the highest used dose of fludrocortisone was 0.5 mg which is considerably higher than recommended.

Malabsorption is a relatively common finding in patients with APECED and it is reported to seriously disrupt the dosing of replacement therapies (7). Thus, malabsorption could affect the electrolyte balance and varying doses of replacement therapies in these patients. We found chronic or episodic diarrhea in most of our patients with hypokalemic episodes. On the other hand, diarrhea was as common in patients who had no tendency for hypokalemia. Malabsorption in patients with APECED is varying in terms of severity and etiology and its diagnosis is difficult to achieve (18). Therefore, it is a potential etiology for electrolyte imbalances even though we could not clearly demonstrate this in our study setting.

In our study, two patients had tubulointerstitial nephritis, which may cause electrolyte disturbances. Both patients had undergone kidney transplantation and the kidney function was followed regularly. Patients with TIN usually present with hyperkalemia instead of hypokalemia (19). However, a recent study showed that patients with APECED who had received kidney transplantation due to TIN experienced electrolyte imbalances mostly due to dehydration or infections. They often required potassium substitution after kidney transplantation (20). Of the two patients with TIN in our study, one was using per oral potassium already at the time of kidney transplantation, but the other patient did not require per oral potassium until shortly after the transplantation. In addition, one patient in our study had been diagnosed with RTA. Hypokalemia is a common finding in patients with RTA (21). Thus, hypokalemia in this patient may be due to the kidney disease.

Hypertension and hypokalemia are classic findings in patients with apparent mineralocorticoid excess (AME) resulting from congenital defect in the function of 11 beta-hydroxysteroid dehydrogenase (22, 23). Thus, we considered altered 11 beta-hydroxysteroid dehydrogenase activity being a potential mechanism for hypokalemia and hypertension. However, we did not find increased cortisol/cortisone ratio or decreased renin concentrations in patients with tendency for hypokalemia, indicating that excess of cortisol does not explain these findings in our patients.

Our findings further indicate that in addition to glucocorticoid and mineralocorticoid deficiency, also adrenal androgen deficiency may be the initial sign of developing adrenal destruction. In our study cohort, three patients without previously diagnosed PAI, had low concentrations of DHEA combined with autoantibodies against 21OH. Two of these patients developed subsequently either glucocorticoid or mineralocorticoid deficiency. This finding of preceding adrenal androgen deficiency has been previously reported in patients with adrenal insufficiency due to defects of hypothalamic-pituitary-adrenal axis and decreased concentrations of ACTH (24). However, in patients with PAI, this has only been hypothesized (25). This finding suggests that in addition to screening for possible glucocorticoid and mineralocorticoid deficiencies, also screening the concentration of DHEA (possibly also its sulphate, DHEAS) may reveal the developing adrenal deficiency in patients with APECED.

This study has some weaknesses. The low number of patients limits the power of analyses in our study. However, the Finnish APECED cohort is one of the largest nationwide cohorts and the records of the patients are comprehensive during the follow-up time of over 50 years. In addition, the retrospective setting of the analysis of hypokalemic patients limits the data that can be comprehensively collected. For example, concentrations of renin were not systematically measured during the hypokalemic periods which could have added important information on the etiologies of hypokalemia. Also, our results do not allow us to provide clear management guidelines. Only a prospective study with fludrocortisone and potassium supplement dosing according to protocol would enable to study the cause relations.

In conclusion, our findings indicate that disturbances in potassium concentrations are found in many patients with APECED with varying frequency, etiology, and severity. Several different etiologies should be taken into consideration when deciding the measures on how to react on imbalanced electrolyte concentrations, but further studies are needed to provide clear management guidelines. In addition, our findings suggest that when screening for possible developing PAI, following the concentrations of adrenal androgens may promote an earlier diagnosis.

## Data Availability Statement

Restrictions apply to the availability of data generated or analyzed during this study to preserve patient confidentiality. The corresponding author will on request detail the restrictions and any conditions under which access to some data may be provided. Requests to access the datasets should be directed to joonatan.borchers@helsinki.fi.

## Ethics Statement

The studies involving human participants were reviewed and approved by Research Ethics Committee of the Hospital District of Helsinki and Uusimaa. Written informed consent to participate in this study was provided by the participants and for subjects aged <18 years, also participants’ legal guardian/next of kin.

## Author Contributions

All authors contributed to the design of the study. JB and SL were responsible for clinical study visits and data collection. JB was responsible for statistical analysis and wrote the first draft of the manuscript. All authors contributed to the interpretation of the results. All authors contributed to manuscript revision, read, and approved the submitted version.

## Funding

This work was supported by The Helsinki University Hospital; The Päivikki and Sakari Sohlberg Foundation; The Finnish Foundation for Pediatric Research; The Finnish Medical Foundation; The Academy of Finland; The Sigrid Jusélius Foundation; The Folkhälsan Research Foundation; The Novo Nordisk Foundation.

## Conflict of Interest

The authors declare that the research was conducted in the absence of any commercial or financial relationships that could be construed as a potential conflict of interest.

The handling editor HF declared a shared affiliation with the author(s) OM at the time of review.

## Publisher’s Note

All claims expressed in this article are solely those of the authors and do not necessarily represent those of their affiliated organizations, or those of the publisher, the editors and the reviewers. Any product that may be evaluated in this article, or claim that may be made by its manufacturer, is not guaranteed or endorsed by the publisher.
